# Soil-based zeolite and metal oxide nanomaterial application alters reactive nitrogen losses and lettuce (*Lactuca sativa* L.) growth

**DOI:** 10.1039/d5en00526d

**Published:** 2025-09-26

**Authors:** Jessica J. Chadwick, Iuliia Mikulska, Aleksandar Radu, Swaroop Chakraborty, Peng Zhang, Sami Ullah, Iseult Lynch

**Affiliations:** a School of Geography, Earth and Environmental Sciences, University of Birmingham Edgbaston Birmingham B15 2TT UK jessicajchadwick@gmail.com i.lynch@bham.ac.uk; b Diamond Light Source Ltd, Harwell Science & Innovation Campus Didcot Oxfordshire OX11 0DE UK; c School of Chemistry, University of Lincoln Lincoln LN6 7TS UK

## Abstract

Excessive nitrogen fertiliser use has resulted in reactive nitrogen losses to the environment through gaseous N emissions, like N_2_O, resulting in agriculture being a major anthropogenic source of N_2_O gas emissions globally. Using engineered nanomaterials to deliver reactive nitrogen can aid in more efficient nutrient delivery to crops, maximising yield and crop quality, while minimising reactive losses to the environment. ZSM-5-15, a nano-zeolite, increased cumulative N_2_O emissions by 134% when applied in combination with a 50% dose of conventional nitrogen fertiliser. This is theorised to be through ion exchange of ZSM-5-15's extra-framework NH_4_^+^ ion load being released, allowing nitrifying microbes to act on the newly released NH_4_^+^ and increase N_2_O emissions. BEA-19, a similar zeolite to ZSM-5-15 but with a slightly altered Si : Al ratio, size and charge, causes no increase in N_2_O emissions. While ZSM-5-15 increases reactive N emissions it also drives improved lettuce growth, with 13% more biomass accumulation compared to a half dose of conventional fertiliser. Ce_0.75_Zr_0.25_O_2_, a nano-metal oxide, improves growth by 6% and maintains the nutritive quality of lettuce, with higher Zn, Cu, Mg, K, Fe and Mn contents, without increasing N_2_O emissions. Nano-Ce_0.75_Zr_0.25_O_2_ transforms in soil to form CeO_2_ and Ce_0.9_Zr_0.1_O_2_, leaching Zr^4+^ ions some of which form ZrCl_4_. These compounds may then act on lettuce roots and soil microbes independently. These results indicate how nanomaterials may impact reactive nitrogen emissions through effects on soil microbial communities.

## Abstract 2

Release of NH_4_^+^ ions from ZSM-5-15 zeolite pore spaces enables plant uptake, microbial immobilization and oxidation to nitrate, where the latter process produces N_2_O gas. Ce_0.75_Zr_0.25_O_2_ NMs transform in the soil, forming ZrCl_4_ and CeO_2_.

## Graphical abstract

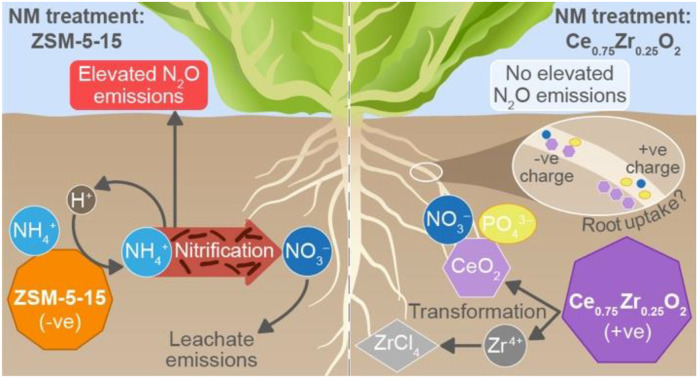

**Environmental significance**

With rising interest in the application of nanomaterials within agriculture, the broader effects of engineered nanomaterials on soils are increasingly of interest. Many nanomaterial studies are performed in pristine environments, however research on nanomaterial mobility and transformation in soil is essential for biologically relevant understanding. This research explores the effects of two soil applied nano-zeolites and a mixed metal oxide on soil N-cycling and lettuce growth as a holistic approach. Research on nanofertilisers often assesses soil functioning indicators like microbial diversity or activity, but lacks consideration of potential impacts on soil nutrient cycling. Here we show that highly differential effects can occur even between nanomaterials of similar compositions and characteristics, and that N-cycling measurements are critical in ensuring safe and sustainable nano-enabled agriculture.

## 1. Introduction

Since the discovery of the Haber–Bosch process in 1913 the total input of reactive nitrogen (Nr) into agricultural ecosystems has more than doubled due to excessive use of synthetic fertiliser.^1^ Increased fertiliser use has supported global food security and human population growth, with over half of the world's current population reliant on nitrogen (N) fertiliser produced food.^2^ Synthetic fertiliser has permitted an increase in crop productivity, low uptake efficiencies from soils (by crops) have enriched agricultural soils with Nr thus resulting in its loss to the environment through aqueous run-off and gaseous emissions. These losses include gaseous N compounds that contribute to climate change, primarily through the production of N_2_O, which has a global warming potential (GWP) of ∼300 as compared to carbon dioxide (CO_2_) and whose atmospheric concentration has increased to 332 ppb in 2021, compared to pre-industrial levels of 275 ppb.^3^ Other volatile N compounds include ammonia (NH_3_) and nitrogen oxides (NO_*x*_), that are formed through different stages of N transformations and contribute to climate change as well as impacting air quality. There are also Nr losses through run-off and groundwater leaching in the form of nitrate (NO_3_^−^) and ammonium (NH_4_^+^), contributing to eutrophication and reducing water quality. It is essential to reduce the impact of synthetic fertiliser on the environment without compromising crop productivity, the ability of global agriculture to support the population, and the livelihoods of farmers.

Nanomaterials (NM) have been posited as a solution for precision agriculture, aiding in efficient nutrient delivery and promoting crop growth and stress tolerance. NMs have at least one dimension between 1 and 100 nm, with a huge surface area to volume ratio permitting environment-dependent transformations (amongst other qualities). NM function is highly dependent on their characteristics, including size, shape, surface charge and chemistry, which in turn alter their reactivity, adsorption/binding kinetics and mobility.^4–6^ The dynamic nature of NMs (as indicated by their propensity to transform) presents a challenge, as generalisations about the behaviour and impacts of different classes of NMs are difficult to make, with changes in shape, size and constituent element ratio altering their fate and effects significantly.^7^ This is particularly true in biological systems. Soil is a highly heterogeneous environment, and NM interactions with minerals, organic matter and microorganisms can trigger ion dissociation and biotransformation of the NMs.^7,8^ Similarly, plants with their varied solute concentrations and highly specialised internal environments, further alters NM transformations, resulting in highly specific interactions between NMs, soil types and plant species.^9,10^ NM introduction to the soil environment causes changes to the physicochemical properties of NMs, altering NM behaviour, fate, and biological activity, with NMs impacted by soil pH, organic matter and water content.^11,12^ These in turn can cause NM agglomeration, transformation, adsorption to the soil matrix or other compounds present and influence NM dissolution, having further impacts on NM behaviour as well as on nutrient supply, including N. Characterising NMs in soil and plants is a further challenge due to the difficulties in tracing the NMs through the whole agricultural system and the requirement that NMs are dispersed for most characterisation methods and in many cases separated from the complex environmental matrix.^13^

Previous research indicates that NM application is able to reduce the nitrogen, phosphorus and potassium (NPK) input needs of crop plants, with metallic NMs shown to impact the cycling of N, P and C.^14,15^ Here, research is focussed on three metallic NMs, two nano-zeolites, BEA-19 and ZSM-5-15, and a mixed metal oxide, Ce_0.75_Zr_0.25_O_2_. Zeolites are highly porous aluminosilicates; their porosity lends itself to specific ion exchange capabilities, making them useful catalysts. In particular, metal-exchanged ZSM-5 NMs have been used in nitrous oxide (N_2_O) decomposition.^16^ Previous research has shown that nanoceria (CeO_2_) and nano-zirconium oxide (ZrO_2_) both have antioxidative properties,^17^ with significant research already exploring the application of CeO_2_ NMs to different crops.^18–20^ The mixed metal oxide utilised here is Ce_0.75_Zr_0.25_O_2_, which follows the nanostructure Ce_1−*x*_Zr_*x*_O_2_. Previous research has utilised Ce_0.75_Zr_0.25_O_2_ NMs as catalysts due to their high dynamic oxygen exchange capacity.^21,22^ This catalytic activity may enable them to function as nanozymes, directly mimicking the action of biological enzymes in the soil to minimise gaseous N emissions.^23^

The experiments presented herein aimed to investigate how soil-based NM application with reduced NPK fertiliser input affected crop growth and yield and soil N gaseous fluxes. The hypothesised mechanism is that binding of the NPK nutrients to the surface of the NMs results in a binding dynamic that allows slow nutrient release and, due to the NMs ability to cross biological barriers and be taken up into plants, would allow a more direct nutrient release, thereby minimising the potential for nutrient losses to the environment. Parameters studied include biomass as a measure of plant growth, antioxidative response to assess NM toxicity or stress initiation to aid in deciphering NM mechanism of action, and elemental analysis in order to determine how NM application altered macro- and micronutrient accumulation in lettuce tissue and if there was above-ground accumulation of the NMs based on analysis of their constituent elements. To understand the nutrient cycling that occurred, CO_2_, N_2_O and NH_3_ gas fluxes were captured *via* gas sampling and analysis. Nitrate, phosphate and ammonium content of the soil and leachate were also analysed to determine how the nutrient supply was influenced by NMs and fertilization. X-ray absorption spectroscopy (XAS) was utilised to determine Ce_0.75_Zr_0.25_O_2_ NM transformations in the soil environment over the timescales of the exposure and uptake by plants. Analysis of the NMs transformations was performed to better understand the mechanisms driving the NMs impacts in the environment and to determine in what form the NMs are when they interact with, or enter, plant tissues. We hypothesized that NM co-application with a reduced dose of fertiliser (half of the conventional dose) would be able to maintain lettuce yield with a reduction in N emissions from soil as a result.

## 2. Materials and methods

### 2.1. Materials

An aqueous dispersion of Ce_0.75_Zr_0.25_O_2_, was obtained from Promethean Particles (UK) *via* the NanoSolveIT project. Powdered ZSM-5-15 (Si/Al = 15.0) and BEA-19 (Si/Al = 19.0) were obtained from Zeolyst (USA). Hydrodynamic diameter and zeta potential were determined using 500 mg L^−1^ NM dispersions in deionised water using a dynamic light scattering (DLS) instrument (Zetasizer, Malvern Instruments, UK). NPK treatment comprised of urea (Sigma-Aldrich) and potassium phosphate monobasic (Sigma-Aldrich). Primary particle size information on BEA-19 was sourced from Jendrlin *et al.*,^24^ on ZSM-5 from Song *et al.*^25^ and on Ce_0.75_Zr_0.25_O_2_ from Dhage *et al.*^26^

### 2.2. Greenhouse study, sampling and analysis

The soil used in the study was collected from FarmED (coordinates 51.869981, −1.581136, https://www.farm-ed.co.uk/) where the loam soil had an oolitic limestone bedrock and a 30 year history of conventional wheat and barley planting. The soil was collected in September 2022 with previous cultivation including barley from March–August 2022. Soils were watered to 60% water holding capacity (WHC) with regular water additions of 100–300 mL per 1.5 kg of soil to maintain soil moisture throughout the experiment. Details of the water chemistry (chemical analysis) are listed in full in the Table S1.

Soils were sieved to 2 mm before 1.5 kg (dry weight) soil was brought back to 60% WHC and left to rest under greenhouse conditions (21 °C, 16:8 light/dark cycle) for one week. Soil pH and electrical conductivity (EC) was recorded before any treatments were applied and the soil was found to be pH 8.1 and 149 μS cm^−1^. After the seven days the soils were treated with either NPK, a combination of half concentration of NPK and NM suspension, or water as a control. In the case of the NM treatments a 25 mg kg^−1^ NM suspension was added to 100 mL of deionised water along with an NPK treatment made from urea and potassium phosphate monobasic. The NM treatment concentration was determined through literature review to be a relatively low NM concentration, minimising the risk of negative ecological impacts, that was still present at sufficient levels to be detectable in the soil.^27^ The full NPK treatment consisted of 180 kg hm^−2^ N, 200 kg hm^−2^ of P_2_O_5_ and K_2_O based on the UK DEFRA RB209A fertiliser manual. The application rates used are recommended for a loamy soil with the previous growing season having been used for winter wheat with an average organic matter content of 3–4%.^28^ The reduced fertilisation was half NPK treatment and consisted of 90 kg hm^−2^ N and 100 kg hm^−2^ of P_2_O_5_ and K_2_O. *Lactuca sativa* L. seeds (“Tom Thumb” variety; Premier Seeds Direct Limited, UK) were sterilised using 1.5% NaClO solution for 10 minutes before rinsing with deionised water until odourless. Five seeds were sown directly in the treated or untreated soils and grown for eight weeks in a greenhouse at 21 °C with 16:8 hour light to dark day cycles. After the first week post-sowing, similarly sized seedlings were maintained (>2 cm tall) and the rest removed from each pot replicate, to leave one seedling per pot. Lettuce growth was monitored through height and width measurements and leaf counts over the course of the eight-week experiment. The lettuce's fresh biomass was weighed at the end of the eight-week growing period before being snap frozen using liquid nitrogen and stored at −80 °C for future analyses.

### 2.3. Soil characterisation and net nitrogen mineralisation

Soils were characterised before and after the 8 week experiment for gravimetric soil moisture, pH and EC. The soil samples were kept after the experiments and stored in a cold room at 4–7 °C. Gravimetric soil moisture was calculated using a 5 g soil subsample that was weighed before and after 24 h drying at 105 °C. Soil extraction for nitrate, ammonium and phosphate analysis was done using 2 M KCl with a 1 : 10 ratio between sieved soil and the KCl solution. The mixture was shaken for one hour at 200 rpm at room temperature before being centrifuged and the supernatant filtered using 0.45 μm syringe filters. The extractant was stored at −20 °C before use. Net nitrogen mineralisation was studied for each biological replicate at the end of the eight-week experiment by incubating 20 g of soil at room temperature in the dark for 28 days before extraction as detailed prior. The post incubation soil extractants were then compared to the end of growth period soil extractions to assess how much nitrogen was nitrified. Dried soil samples underwent elemental analysis and were digested for analysis by inductively coupled plasma optical emission spectroscopy (ICP-OES) as described below.

### 2.4. Greenhouse gas fluxes

Static chamber gas sampling with gas chromatography was performed to measure N_2_O and CO_2_ emissions. Static chambers had dimensions 15 × 20 × 20 cm and were sealed at the base with water to ensure they were airtight. 15 mL of gas samples were taken at 0 hours, 0.5 hours, 1 hour and 2 hours on a weekly basis and stored in 12 mL pre-evacuated exetainer vials (Labco Limited, UK). The gas samples were analysed using the Agilent 7890A Gas Chromatograph (GC) interfaced with a PAL3 autosampler (Agilent Technologies Ltd, USA) following the method used in Comer-Warner *et al.*^29^ and using reference standards as described in Sgouridis and Ullah.^30^ Every 20 samples a standard (N_2_O = 0.2 ppm, CH_4_ = 4 ppm, and CO_2_ = 500 ppm) was used to prevent drift with minimum detectable concentration differences of 9 ppb N_2_O, 72 ppb CH_4_ and 31 ppm CO_2_. GC analysis of N_2_O used a micro electron capture detector (μECD), while a flame ionisation detector (FID) was used to analyse CH_4_ and CO_2_ methanised into CH_4_.

A 1 M H_2_SO_4_ solution with 2% (w/v) glycerol was used as an acid trap for ammonia emissions from the soils. The acid trap was incubated inside the static chambers for two hours during the weekly gas sampling. The acid trap solution was then extracted with deionised water before NH_4_^+^ analysis *via* the ISO/DIS 15923-1 standard analysis method on an AQ400 Discrete Analyser (SEAL Analytical, WI, USA).

### 2.5. Soil and lettuce analyses

#### 2.5.1 Soil nutrient analysis

Soils in the pots containing the growing lettuce plants were watered with 100–300 mL per pot to reach 60% WHC, and a funnel was used to capture the first 50 mL of resulting leachate on weeks 1, 2, 4 and 8. The funnel was washed with deionised water between replicates and treatments. This leachate was filtered using a 0.45 μm syringe filter and stored at −20 °C until preparation for analysis within a week of sampling date. Leachate and KCl extracted soil samples were analysed for nitrate, ammonium and orthophosphate concentration using a Tecan Spark microplate reader (Tecan, UK). Ammonium analysis was performed in accordance with the Mulvaney^31^ protocol. Samples and reagents for the ammonium assay were incubated at 40 °C for 15 minutes, returned to room temperature, and absorbance readings were taken at 650 nm. Nitrate concentrations were determined using a VCl_4_ based method.^32^ Microplates underwent a 6–12 hour incubation at 4 °C before an absorbance reading was taken at 540 nm. The phosphate assay was performed as described by Murphy and Riley.^33^ The microplate was incubated for 10 minutes at room temperature before taking an absorbance reading at 880 nm. Each assay used their respective standard solutions which were made up in the same extractant background as the samples, KCl or tap water.

#### 2.5.2 Macro- and micronutrients, NM constituent elements and plant stress marker analysis

Samples (lettuce and soil) were digested for inductively coupled plasma optical emission spectroscopy (ICP-OES) according to the protocols for spinach tissue and the US EPA 3052 protocol for soil/sediment as described in the MARS 6 Microwave Acid Digestion Method Note Compendium.^34^ Digestion program details are displayed in full in Tables S2 and S3. Resulting digestate was diluted 50-fold before running samples on the Perkin Elmer optima 8000 ICP-OES for analysis (Agilent Technologies Ltd, USA). The elements studied were P, K, Zn, Ca, Mg, Fe and Mn, as well as the NM constituent elements Ce, Zr, Al and Si. Both dried soil samples and freeze-dried lettuce tissue samples underwent elemental analysis (EA) for C, N, and S. EA details are elaborated on in the SI. Frozen lettuce tissues were used for a malondialdehyde (MDA) assay which measures lipid membrane peroxidation as a marker for oxidative stress (Sigma-Aldrich, UK). The assay was performed according to the manufacturer's instructions.

### 2.6. X-ray absorption spectroscopy

Soil samples spiked with 3000 mg kg^−1^ Ce_0.75_Zr_0.25_O_2_ NMs were used for X-ray absorption spectroscopy (XAS) analysis. Soils were treated with the NMs dispersion and mixed thoroughly, followed by 8 weeks of incubation at 21 °C. 200 mg soil samples were pelleted for analyses, with XAS spectra collected on beamline B18 (ref. 35 and 36) at the Diamond Light Source synchrotron radiation facility (Didcot, UK). Zr K-edge XAS spectra on soil samples were collected in fluorescence mode using a Canberra 36-pixel monolithic segmented hyper pure germanium detector (HPGe) with Xspress4 signal processing,^37^ while Ce L_III_-edge spectra were collected in fluorescence mode using Vortex-ME4 silicon drift detector partnered with the Xspress3 digital pulse processor. Details of XAS spectra collection for experimental samples, reference compounds and reference foils are in the SI. Demeter software package was used to perform the data analysis, including energy calibration, normalisation and linear combination fitting (LCF) analysis.^38^

### 2.7. Statistics

R Statistical Software was used for all analyses.^39^ Data was initially assessed for normality using Shapiro–Wilk tests, *Q*–*Q* plots and homogeneity of variance testing. Where normality was breached, non-parametric statistical analyses were performed. Kruskal–Wallis tests, followed by Dunn's *post hoc* test for pairwise comparison were performed for the soil samples. Normally distributed data were analysed with one-way ANOVA, followed by *post hoc* Tukey HSD (honest significant difference) tests. GC calculated N_2_O and CO_2_ concentrations, and leachate nutrient concentrations, were assessed using linear mixed models to interrogate treatment and time effects on emissions. *Post hoc* comparisons of linear mixed model results were evaluated using Tukey tests. Lettuce biomass comparisons were performed using Bonferroni corrected two-sample *t*-tests comparing control lettuce with all other treatments. The threshold for statistical significance was set at *p* = 0.05.

## 3. Results

500 mg L^−1^ NMs dispersed in water showed both zeolites as negatively charged (Table 1) with BEA-19 having a more negative zeta potential than ZSM-5-15. The Ce_0.75_Zr_0.25_O_2_, however, was positively charged. The three pristine NMs also had varying agglomerate sizes as determined *via* DLS, with BEA-19 agglomerates the largest and Ce_0.75_Zr_0.25_O_2_ NMs the smallest.

**Table 1:** Nanomaterial characterisation using dynamic light scattering (DLS) before exposure to soil. Data are means ± standard deviation

Nanomaterial	Mean hydrodynamic size (nm) (*n* = 3)	Mean zeta potential (mV) (*n* = 3)
BEA-19	997.8 (± 196.8)	−39.5 (± 0.95)
ZSM-5-15	631.3 (± 12.7)	−28.0 (± 0.66)
Ce_0.75_Zr_0.25_O_2_	104.5 (± 4.37)	+45.2 (± 0.75)

BEA-19 primary particle size was found to be 0.05 μm, as measured by SEM or TEM.^24^ According to Jendrlin *et al.*,^24^ ZSM-5 particles were 0.2 μm. Another paper found Zeolyst sourced ZSM-5-15 particles to be 32 nm, with larger aggregates found between 700–1000 nm (25). Ce_0.75_Zr_0.25_O_2_ primary particle size was found to be 5 nm using TEM.^26^ The SEM and TEM derived particle sizes makes it clear that the NMs are agglomerated, and that the hydrodynamic size values are thus for the NM aggregates.

Soil moisture varied across treatments, with the control soil having greater moisture levels than BEA-19 (*p* = 0.02), Ce_0.75_Zr_0.25_O_2_ (*p* = 0.0041), and ZSM-5-15 (*p* = 0.00061) treated soils. Additionally, soil moisture was significantly lower in ZSM-5-15-treated soils than that of NPK full (*p* = 0.01) (Table 2). Soil pH varied across treatments, with NPK full soil pH being significantly lower than in Ce_0.75_Zr_0.25_O_2_ treated soil (*p* = 0.0052) or NPK half treated soil (*p* = 0.01). The positively charged Ce_0.75_Zr_0.25_O_2_ NMs had a particularly alkalinizing effect on the soil, producing a significantly higher soil pH than in the control (*p* = 0.032). Electrical conductivity (EC) of the soil was also affected by the various treatments. The EC of NPK full treated soil was significantly higher than that of the control soil (*p* = 0.028) or ZSM-5-15 treated soil (*p* = 0.0057).

**Table 2:** Soil properties in the six different soil treatments after the eight-week growth period. Data are means ± standard error. Absent data is due to values being below the limit of detection of the specific method. NPK is the standard recipe of the synthetic fertilizer, nitrogen, phosphorus, potassium, and half NPK is half of the standard dosage

Soil properties	Control	NPK full	NPK half	BEA-19 + NPK half	Ce_0.75_Zr_0.25_O_2_ + NPK half	ZSM-5-15 + NPK half
Gravimetric soil moisture (%) (*n* = 4)	73.9 (± 1.1)	69.9 (± 2.9)	66.3 (± 1.6)	63.1 (± 1.5)	60.9 (± 1.2)	58.2 (± 3.2)
pH (*n* = 4)	8.31 (± 0.06)	6.84 (± 0.18)	8.82 (± 0.05)	8.65 (± 0.07)	8.86 (± 0.04)	8.60 (± 0.09)
EC (μS cm^−1^) (*n* = 4)	49.5 (± 3.3)	76.4 (± 8.5)	52.1 (± 2.6)	60.0 (± 10.5)	46.2 (± 4.9)	39.5 (± 2.0)
NO_3_^−^ (mg kg^−1^ dry soil) (*n* = 4)	2.81 (± 0.43)	9.14 (± 2.59)	4.02 (± 1.15)	5.70 (± 2.73)	3.29 (± 1.09)	1.82 (± 0.39)
Post-incubation NO_3_^−^ (mg kg^−1^ dry soil) (*n* = 4)	0.789 (± 0.33)	11.7 (± 1.85)	3.28 (± 1.54)	4.36 (± 2.06)	1.80 (± 0.68)	0.934 (± 0.056)
PO_4_^3−^ (mg kg^−1^ dry soil) (*n* = 4)	—	0.01 (± 0.0041)	0.0017 (± 0.0007)	0.00081 (± 0.0002)	0.00065 (± 0.00024)	0.0012 (± 0.00026)
N (%) (*n* = 4)	0.26 (± 0.01)	0.28 (± 0.02)	0.28 (± 0.01)	0.28 (± 0.01)	0.27 (± 0.004)	0.28 (± 0.01)
C (%) (*n* = 4)	5.89 (± 0.34)	5.89 (± 0.41)	6.28 (± 0.02)	5.98 (± 0.13)	6.07 (± 0.02)	6.02 (± 0.21)
C : N (*n* = 4)	22.8 (± 0.3)	20.9 (± 0.7)	22.3 (± 1.0)	21.8 (± 0.7)	22.5 (± 0.6)	21.4 (± 0.8)
K (mg kg^−1^ dry soil) (*n* = 4)	8469 (± 311)	8396 (± 368)	7913 (± 257)	7838 (± 254)	9734 (± 1147)	8497 (± 268)
P (mg kg^−1^ dry soil) (*n* = 4)	1170 (± 85)	1270 (± 93)	1158 (± 39)	1105 (± 26)	1364 (± 93)	1095 (± 55)
Cu (mg kg^−1^ dry soil) (*n* = 4)	26.0 (± 6.7)	15.8 (± 1.9)	25.7 (± 6.7)	55.4 (± 3.7)	43.4 (± 1.5)	84.8 (± 7.3)
Al (mg kg^−1^ dry soil) (*n* = 4)	18 916 (± 416)	17 482 (± 339)	17 962 (± 613)	15 010 (± 585)	21 291 (± 4932)	16 687 (± 291)
Si (mg kg^−1^ dry soil) (*n* = 4)	170 418 (± 7860)	160 528 (± 13 517)	151 159 (± 8819)	146 620 (± 5459)	156 984 (± 7853)	173 300 (± 11 333)
Ce (mg kg^−1^ dry soil) (*n* = 4)	15.7 (± 3.8)	15.5 (± 3.9)	10.3 (± 1.0)	12.0 (± 2.6)	14.4 (± 2.5)	13.3 (± 3.0)
Zr (mg kg^−1^ dry soil) (*n* = 4)	30.4 (± 2.7)	22.9 (± 4.1)	22.2 (± 2.6)	23.3 (± 3.8)	33.6 (± 10.8)	39.2 (± 5.9)

No meaningful differences were found between soil NO_3_^−^ or PO_4_^3−^ concentrations across treatments, with control soil having an undetectable PO_4_^3−^ concentration (Table 2). There was a disparity between the concentrations of NO_3_^−^ and post-incubation NO_3_^−^ under NPK full treatment, indicating that N was nitrified in this treatment only. The full NPK treatment resulted in significantly more mineralised NO_3_^−^ compared to all other treatments (Table S4). C : N ratio varies across treatments, with no statistically significant deviation from the control across treatments for C : N ratio, or C and N content alone. The non-statistically significant differences seen between NO_3_^−^ concentrations differ from the consistent overall N content of the soil due to nitrate making up only a small proportion of total N compounds in the soil.

Comparing the elemental concentrations for macro- and micronutrients across the treatments reflects broadly similar nutrient profiles (Table 2). The only element to have significantly different elemental concentrations was Cu. ZSM-5-15 treated soils had much higher Cu concentrations, with more than 5× the amount of Cu present in soil as compared to the NPK full treated soil (*p* = 0.007). The Cu content of soil in the BEA-19 treatment was also significantly different to NPK full (*p* = 0.26). Soil nanomaterial constituent concentrations were uniform across treatments other than for Al. BEA-19 Al soil content was significantly lower than that of the control soil (*p* = 0.0084).

**Fig. 1:**
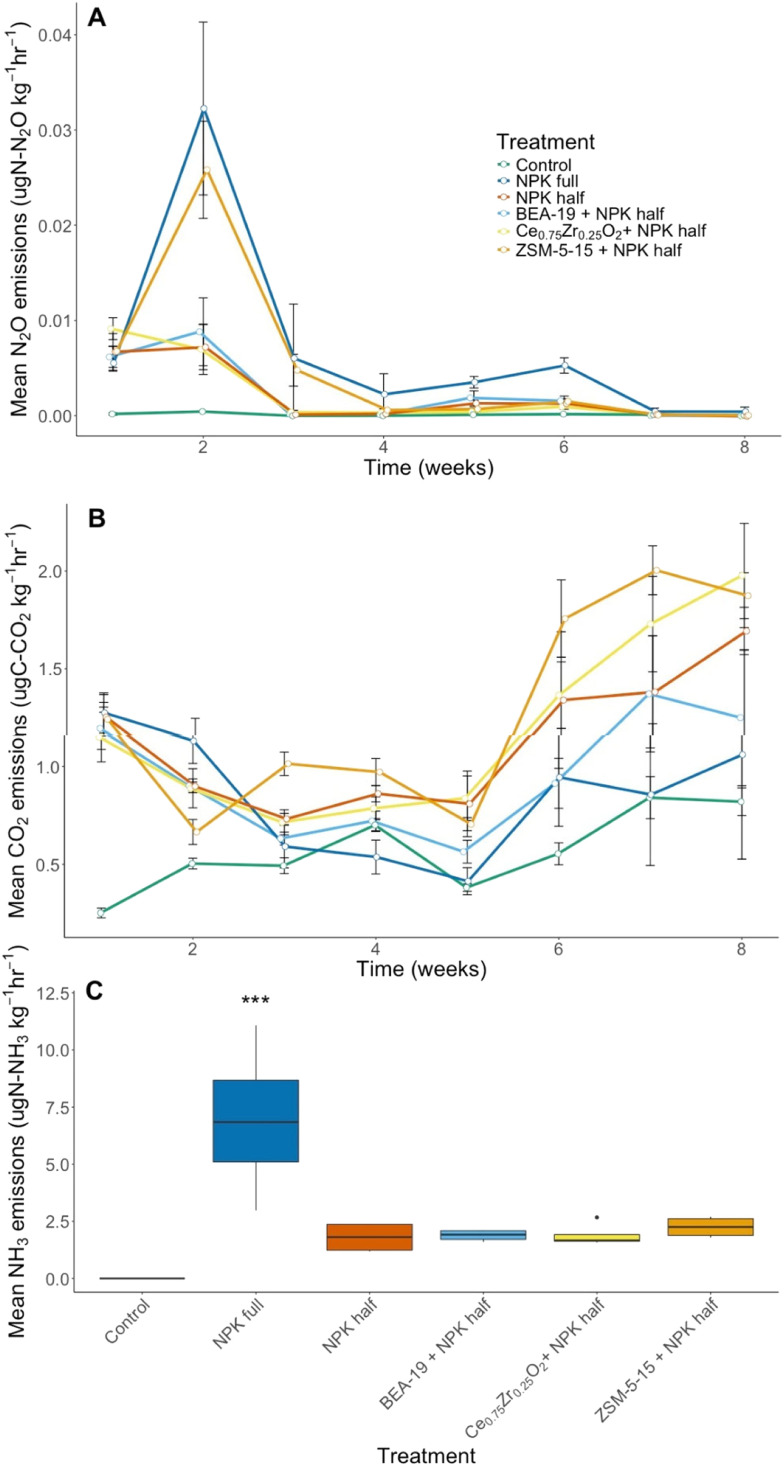
(A) Weekly time course of soil N_2_O emissions across the five different treatments and control. Error bars indicate standard error from the mean (SEM) based on 4 replicates. (B) Weekly time course of soil CO_2_ emissions across the five different treatments and control. Error bars indicate SEM of 4 replicates. (C) Soil NH_3_ gas emissions in week 1 of the experiment. Error bars indicate SEM from 4 replicates. “***” is used to denote a statistically significant result as compared to the control of *p* < 0.0001.

NPK full treatment produced the greatest N_2_O emissions in week 2 (Fig. 1A), but also cumulatively (Fig. S1A). The NPK half combination treatments were consistently low producers of N_2_O with one notable exception. Co-addition of ZSM-5-15 NM with NPK half produced 42% more N_2_O over the course of the 8 week period as compared to NPK half addition alone. This is most pronounced in week 2, as with the NPK full N_2_O emissions. N_2_O emissions are significantly different across exposure weeks (*p* = 2.58 × 10^−10^). NPK full N_2_O emissions are significantly different to all treatments (*p* < 0.05) other than ZSM-5-15 (*p* = 0.793). ZSM-5-15 produces elevated emissions, producing significantly more N_2_O emissions than the control soil (*p* = 0.0165).

CO_2_ evolution was used as a proxy for soil respiration (Fig. 1B), with cumulative CO_2_ produced from each treatment also calculated (Fig. S1B). Initially, CO_2_ evolution reflects a similar trend to N_2_O emissions, with NPK full treatment having the greatest emissions at weeks 1 and 2, as seen in Fig. 1B. Towards the end of the 8 week growing period CO_2_ emissions are elevated above week 1 CO_2_ emissions, with an associated increase in respiratory activity, from ZSM-5-15, Ce_0.75_Zr_0.25_O_2_ and NPK half treatments. Week of exposure had a significant effect on CO_2_ emissions captured (*p* = 1.47 × 10^−8^). Control CO_2_ emissions were significantly different compared to all other treatments (Table S6), other than NPK full application (*p* = 0.0785).

Analysis of NH_3_ gas emissions was performed for all 8 weeks of growing, however, results greater than the limit of detection were only found for week 1 of the experiment (Fig. 1C). Comparison of NH_3_ gas emissions between treatments showed that NPK full and control soil were statistically significantly different (*p* = 0.0000323). Emission factors of NH_3_ and N_2_O emissions are available in Fig. S2.

**Fig. 2:**
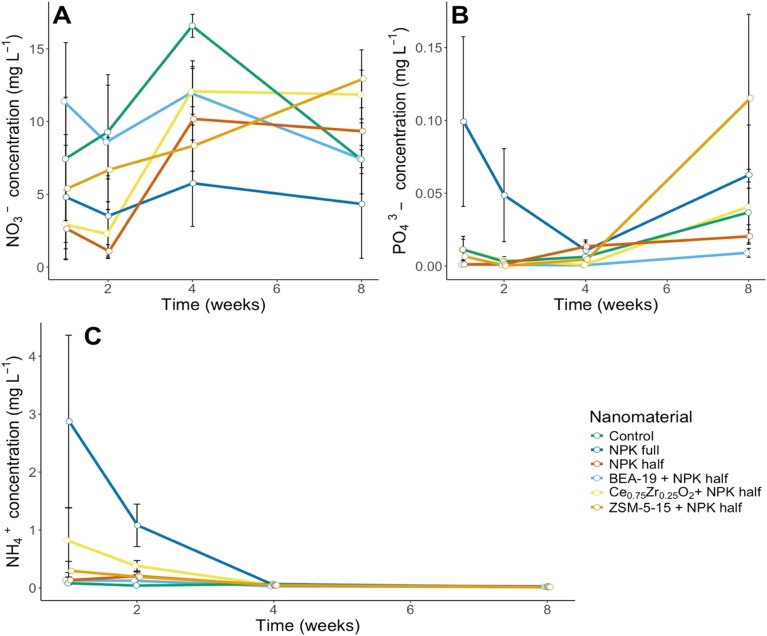
(A) NO_3_^−^ concentration in leachate emissions recorded at weeks 1, 2, 4, and 8 across the six treatments. (B) PO_4_^3−^ concentration in leachate across weeks 1, 2, 4 and 8. (C) NH_4_^+^ concentration in leachate recorded at weeks 1, 2, 4 and 8. Error bars for A–C reflect SEM based on 4 replicates.

NO_3_^−^ losses in leachate peaked for all treatments at week 4, as shown in Fig. 2A, other than for the ZSM-5-15 treated soil, which peaked in week 8 with 12.9 mg L^−1^ of NO_3_^−^ in the leachate. This is not the highest NO_3_^−^ leachate concentration overall, which is found at week 4 in the control soil (16.6 mg L^−1^). NPK full has the lowest NO_3_^−^ emissions overall. All treatments, other than BEA-19, generated lower NO_3_^−^ leachate concentrations than the control (Table S5). Exposure duration (week) and treatment have a significant effect on the NO_3_^−^ emissions arising from the Ce_0.75_Zr_0.25_O_2_ NM treatment (*p* = 0.013), with exposure duration (week) also proving significant for NPK half (*p* = 0.034) and ZSM-5-15 (*p* = 0.046) treated soil NO_3_^−^ concentrations.

The PO_4_^3−^ concentration in the soil was much higher for NPK full than for all the other treatments (0.099 mg L^−1^), as seen in Fig. 2B. A statistically significant difference was found between the PO_4_^3−^ soil concentration of NPK full and control treatments (*p* = 0.00633). There was a minor reduction in PO_4_^3−^ emissions from Ce_0.75_Zr_0.25_O_2_ and ZSM-5-15 treatments relative to the half NPK treatment, this difference, however, was not statistically significant. ZSM-5-15 treatment is significantly correlated with exposure duration (week) (*p* = 0.0053).

NH_4_^+^ concentration in leachate peaked in week 1 before rapidly decreasing, with NPK full emissions reducing by 1 mg L^−1^ per week until levels stabilise from week 4 (Fig. 2C). NH_4_^+^ emissions from the NPK full treated soil are significantly higher than the control across all timepoints (*p* = 2.48 × 10^−6^) and are significantly correlated with treatment duration (week) (*p* = 0.00075), as is evident from Fig. 2C. NPK full treatment NH_4_^+^ emissions are also significantly different to BEA-19 (*p* = 0.0036), NPK half (*p* = 0.0048) and ZSM-5-15 treatment emissions (*p* = 0.0079). The lack of significant differences between Ce_0.75_Zr_0.25_O_2_ and NPK full treatments is indicative that while the increase in emissions under Ce_0.75_Zr_0.25_O_2_ treatment is not different to the other treatments, it is still elevated.

**Fig. 3:**
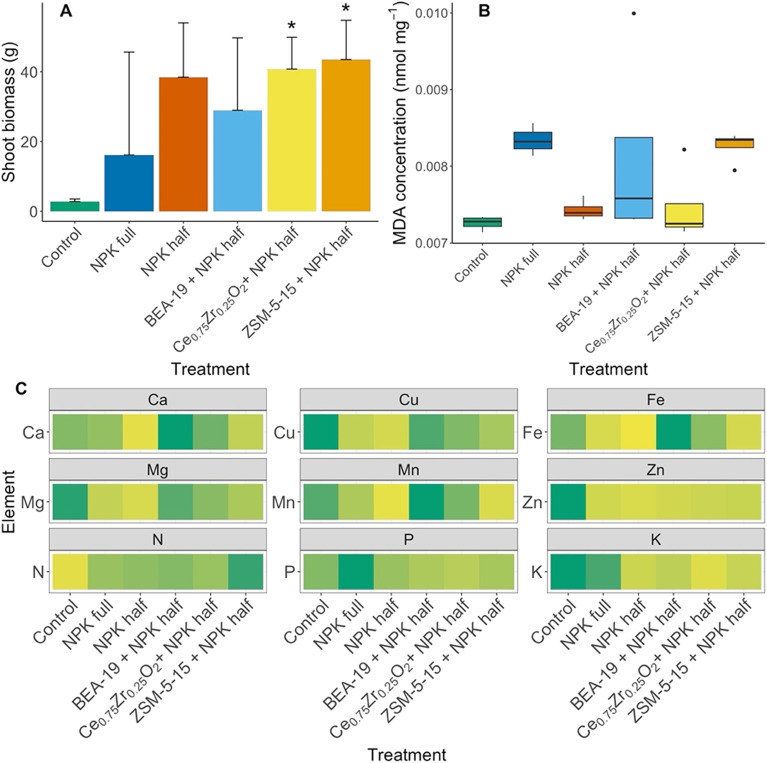
A) Lettuce aboveground biomass after destructive sampling at week 8. Error bars indicate standard deviation (*n* = 4). Significantly different means compared to the control are denoted using ‘*’ where *p* < 0.05. B) Malondialdehyde (MDA) concentration in lettuce shoot tissue including mean and SEM (*n* = 4). Black dots signify outliers. C) Relative concentration of macro- and micronutrient elements of interest in lettuce tissue compared across the six treatments (*n* = 4), numerical element concentration data is available in Table S7. N content was determined *via* elemental analysis, all other nutrients were determined *via* ICP-OES.

The highest lettuce yields were produced under Ce_0.75_Zr_0.25_O_2_ (40.8 g) and ZSM-5-15 (43.5 g) treatments, with NPK half treatment alone producing on average 38 g lettuce, as shown in Fig. 3A. Significant increases compared to the control lettuce yield were found under ZSM-5-15 (*p* = 0.027) and Ce_0.75_Zr_0.25_O_2_ treatment (*p* = 0.017). Images of the lettuces before harvest at week 8 are provided in Fig. S3.

MDA concentration is a measure of lipid peroxidation, or the effect of ROS on cell membranes. Control lettuce had the lowest MDA concentration of 0.0073 nmol mg^−1^ of lettuce tissue (Fig. 3B). Similarly, NPK half, BEA-19 and Ce_0.75_Zr_0.25_O_2_ treated lettuces didn't show the presence of the stress marker, although variability in the BEA-19 data was large. MDA levels were elevated under NPK full and ZSM-5-15 treatments, but these were not found to be statistically significant increases in MDA concentration relative to the control lettuce.

The micronutrient tissue concentrations, apart from Zn, appear to show similar trends (Fig. 3C). Control tissues have higher concentrations of Cu, Mn and Mg (*p* < 0.05) compared to all other treatments. BEA-19 treated lettuce had a lower yield than controls and it can be seen to have higher micronutrient concentrations of Ca, Cu, Fe, Mn and Mg. There were no statistically significant differences found between treatments for any other micronutrients other than Zn. N content of ZSM-5-15 grown lettuce was significantly greater than that in the control lettuce (*p* = 0.0067). The P content of NPK full treated lettuce was greater than that in control (*p* = 0.022), BEA-19 (*p* = 0.012), or Ce_0.75_Zr_0.25_O_2_ lettuce (*p* = 0.043). Ce_0.75_Zr_0.25_O_2_ lettuce had significantly lower K content than the control (*p* = 0.038) or NPK full (*p* = 0.013) lettuce. Differences in elemental concentrations of NM constituent elements in lettuce tissues can be found in Fig. S4.

**Fig. 4:**
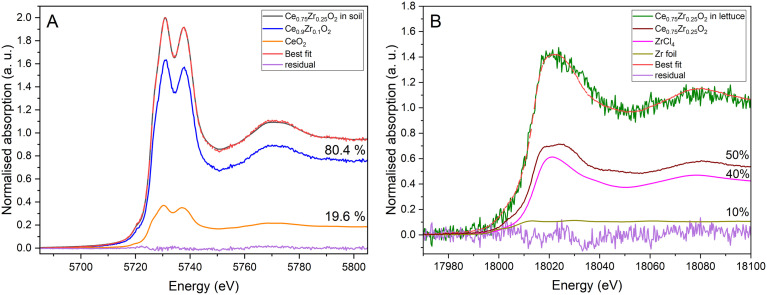
(A) Ce L_3_-edge normalised XANES spectrum of Ce_0.75_Zr_0.25_O_2_ treated soil samples and best linear combination fit of XANES profiles of Ce_0.9_Zr_0.1_O_2_ and CeO_2_. (B) Zr K-edge normalised XANES spectrum of Ce_0.75_Zr_0.25_O_2_ grown lettuce tissue and best linear combination fit of XANES profiles of pristine Ce_0.75_Zr_0.25_O_2_ and ZrCl_4_ and Zr metal foil.

The Ce fraction of the NM is completely transformed from the original Ce_0.75_Zr_0.25_O_2_ in the soil. The linear combination fit (Fig. 4A) is able to fully describe the transformation of the NM in soil using the X-ray absorption near edge structure (XANES) spectra of CeO_2_ (19.6% ± 0.8%) and Ce_0.9_Zr_0.1_O_2_ (80.4% ± 0.8%). The results of the linear combination fit were not able to fully reproduce the Zr K-edge experimental spectra for the Ce_0.75_Zr_0.25_O_2_ treated soil, however the Zr K-edge XANES spectrum for the Ce_0.75_Zr_0.25_O_2_ grown lettuce tissue shows that Ce_0.75_Zr_0.25_O_2_ is translocated into aboveground tissues as well as Zr metal and ZrCl_4_. Reference standards and experimental spectra are presented in Fig. S5.

## 4. Discussion

### 4.1. N_2_O emissions and soil respiration are elevated under ZSM-5-15 treatment

ZSM-5-15 treatment both elevates N_2_O emissions and increases the soil respiration rate. The observed peak in N_2_O emissions at week-2 is due to a lag in microbial gas production after the immediate addition of nutrients. N was added to soil in the form of urea which is hydrolysed to NH_4_^+^*via* the enzyme urease, which is then utilised in nitrification, producing N_2_O. Nitrate can also be denitrified into N_2_O in addition to nitrification as a source of N_2_O. Zeolite application has been shown to increase the abundance of ammonia-oxidising archaea and to increase ammonia monooxygenase enzyme activity in agricultural waste.^40^ ZSM-5-15 may therefore be impacting nitrifying microbes and elevating N_2_O emissions either through increasing nitrifying community size, or directly impacting on enzymatic activity. Soil pH was reduced under the NPK full treatment due to the increased addition of N fertiliser triggering subsequent nitrification, producing H^+^ ions, as seen in eqn (1), which shows the *Nitrosomonas* catalysed reduction of NH_4_^+^ in soil. This correlates with the reduction in NH_3_ gas emissions and NH_4_^+^ in the leachate. However, there is no reduction in soil pH under the ZSM-5-15 treatment. If the theorised mechanism is that ZSM-5-15 is able to upregulate nitrification, then, as with the NPK full treatment, there should be a corresponding drop in soil pH. The absence of a pH change may mean that ZSM-5-15 has a buffering capacity, potentially through its extra-framework ion (NH_4_^+^) being released, leaving binding sites for H^+^ ions through a process of ion exchange.^41,42^ Extra-framework ions are held in the pore spaces of the zeolites' 3D structure and are not chemically bound to the NM, allowing greater capacity for ion exchange than other comparable minerals.^43^

1 2NH_4_^+^ + 4O_2_ + 4e^−^ → 2NO_3_ + 4H^+^ + 2H_2_O + 12e^−^

Alternatively, the ZSM-5-15 triggered N_2_O emissions could be through an effect on the final stage of denitrification. This might be through ZSM-5-15 having a toxic effect on soil microbes that are responsible for reducing N_2_O to N_2_, causing there to be a proportional increase of N_2_O. Nitrous oxide reductase (NOS) is the only enzyme found to reduce N_2_O to N_2_ in the N cycle.^44^ It may be that ZSM-5-15 has an inhibitory effect on this enzyme, producing elevated N_2_O emissions, however, this theory doesn't align with the soil respiration data or the leachate emissions data.

The increase in soil respiration, as determined *via* CO_2_ production, cannot definitively be determined to be produced through either an increase in lettuce root biomass, impacts on the soil microbial community, or both. Previous research shows that the metabolic quotient of soil increased under zeolite application although no impact on respiratory activity was seen.^45^ Due to the sampling procedure utilised, no accurate data for root biomass was found in this study, leaving the mechanism unclear.

ZSM-5-15, nor either of the other NM treatments produced any impact on NH_3_ emissions, with the highest emissions being from NPK full application. Zeolite addition to poultry manure has been shown to reduce NH_3_ volatilisation by up to 44%.^46^ While the organic matter content of manure is much higher than that of soil, the ability to reduce emissions is relevant. The low NH_3_ emissions may be due to the rate of nitrification in the ZSM-5-15 treated soil, whereby NH_3_ is being converted rather than emitted as a gas.

Rather than promoting microbial community shifts, ZSM-5-15 may have nanozyme activity and could be directly involved in conversion of NH_4_^+^ to N_2_O. Other zeolite nanozymes have been developed previously, but were functionalised with Zn.^47,48^ As ZSM-5-15 has no transition metal functionalisation, these findings are indicative that ZSM-5-15 is able to produce a shift in typical terrestrial N cycling, as compared to control or NPK half treatment alone, likely by promoting nitrifying microbe abundance or enzymatic activity.

### 4.2. Leaching loss dynamics over time and with NM treatment

The timing and magnitude of nutrient emissions in leachate is consistent with the gas emissions data for ZSM-5-15 treatment and its effect on nitrification sourced N_2_O emissions. In week 1, the lowest NO_3_^−^ concentration in the leachate was for Ce_0.75_Zr_0.25_O_2_ treatment, whilst NO_3_^−^ concentrations were highest in the control soil. An explanation for this could be due to the NPK half addition producing a lag in emissions as seen with N_2_O, due to urea's conversion by urease to NH_4_^+^ before conversion to NO_3_^−^. The peak in ammonium concentration in week 1, and decline by week 4, coincides with the peak in nitrate emissions in leachate. The control soil had the lowest N_2_O emissions, as compared to the NPK full and all NM with half NPK treatments. This could be due to nitrification of NH_4_^+^ to NO_3_^−^ occurring more completely, with reduced nitrifier sources of N_2_O. Both zeolites used in this experiment had NH_4_^+^ as their extra-framework ions. This binding capacity for NH_4_^+^ means that there may be a delay in nitrification under this treatment due to slow release of the NH_4_^+^. Overall, this will lower the amount of nitrification in the soil at later stages due to the binding of NH_4_^+^. The increase in leachate concentration of NO_3_^−^ over time for the zeolites indicates a slow release of these extra-framework ions, with BEA-19 potentially having a stronger affinity for NH_4_^+^ than ZSM-5-15. BEA and ZSM-5 type zeolites have been compared for ion exchange application in catalytic converter exhaust gas adsorption, reflecting the fact that both have catalytic and adsorptive effects across applications.^49^ This also has a further impact on denitrification, due to low nitrification later in the growth period limiting the supply of NO_3_^−^ to denitrifying microbes.

NO_3_^−^ leachate emissions are highest for the control group, peaking in week 4, this is partnered with the lowest rate of CO_2_ evolution, indicating that denitrification is stunted in the control soils through a lack of soil microbial activity. This reduced microbial activity, combined with the lack of NPK input to the soil, causes the low N_2_O emissions. The NO_3_^−^ emissions for NPK full treatment are likely so low due to the soil pH reduction impacting on soil microbial activity, as displayed through the reduced CO_2_ emissions data. This lack of activity slows the N cycle, with reduced NH_4_^+^ and NO_3_^−^ emissions. NPK half and Ce_0.75_Zr_0.25_O_2_ treatments follow the same trend for NO_3_^−^ emissions; Ce_0.75_Zr_0.25_O_2_ produces slightly elevated emissions but this is coupled with greater soil respiration. NO_3_^−^ emissions for ZSM-5-15 are unique, peaking in week 8, reflecting the time period needed for conversion of NH_4_^+^ to NO_3_^−^, further indicating that the earlier (week 2) N_2_O emissions peak is nitrification sourced rather than arising from denitrification.

Leachate losses over time reflect changing adsorption dynamics and local conditions for the NMs, with the highest PO_4_^3−^ emissions occurring in ZSM-5-15 treated soil at week 8. ZSM-5-15 is negatively charged and so, unlike Ce_0.75_Zr_0.25_O_2_, there are no binding dynamics between it and PO_4_^3−^, thus increasing PO_4_^3−^ emissions. BEA-19 however has an even more negative zeta potential. Zeolites have previously been utilised for simultaneous removal of NH_4_^+^ and PO_4_^3−^ in water purification, using Ca^2+^ ions to remove PO_4_^3−^.^50^ As the extra-framework ion, NH_4_^+^, is slowly released over time, pore spaces in the structure of the zeolite will become available, permitting ion exchange. This may happen with H^+^ ions as mentioned previously but also other cations, for example Ca^2+^, K^+^ and Na^+^. As ion exchange continues to occur this may result in formation of Ca_3_(PO_4_)_2_, minimising PO_4_^3−^ leaching under BEA-19 treatment. While both BEA-19 and ZSM-5-15 are negatively charged nano-zeolites with relatively similar Si : Al ratios (19 and 15, respectively), pore sizes (6.8 Å and 5.5 Å) and primary particle sizes, they have very different impacts on gaseous and leachate emissions, and final soil concentrations of NO_3_^−^ and PO_4_^3−^, however the physical basis for these differences is currently unknown.

The leachate emissions data, combined with the N_2_O and CO_2_ emissions in particular, have a shared narrative around the impact of ZSM-5-15 NMs on soil nitrification. Differences between the NMs, both in terms of constituent elements and hydrodynamic size, zeta potential and other properties, are likely significant in understanding the mechanisms that impact their leachate emissions.

### 4.3. NM zeta potential, ion exchange capacity, and transformations within the soil matrix, influence NM–soil component interactions

Comparing SEM/TEM derived particle sizes and water-dispersed hydrodynamic sizes it is clear that the NMs exist as aggregates under the exposure conditions. Further agglomeration and transformation will then have altered the NMs before interaction with plant roots, altering their capacity for translocation into other plant tissues. The XANES data reflects that the NMs are undergoing transformations within the soil environment. Ce and Zr were below the limit of detection for ICP-OES in lettuce tissue, while this has meant a high quality XANES spectra was not able to be generated for Ce, indicating limited bioaccumulation. In order to understand if the small amounts of translocated NMs undergo further transformations in plant tissues, studies at higher, less environmentally realistic, concentrations are required. The LCF for Ce L3-edge XANES spectra of Ce_0.75_Zr_0.25_O_2_ in soil clearly shows Zr^4+^ ions may leach from the mixed metal oxide, leaving Ce_0.9_Zr_0.1_O_2_ and CeO_2_ in the soil. Previous study shows that CeO_2_ has limited effect on soil microbial biomass, however there may be uptake of Ce by soil microbes.^51^ This indicates one of the ways in which CeO_2_ can interact with the biotic components of soil. Ce_0.75_Zr_0.25_O_2_, ZrCl_4_ and Zr metal were the primary forms of Zr found in the lettuce samples after the 8 weeks of incubation. Whether the leached Zr^4+^ ions are forming ZrCl_4_ in the soil or inside the plant roots/shoots is unclear due to low quality soil spectra. ZrCl_4_ is used industrially and as a catalyst, however ZrO_2_ is the more popular nano-form for application. ZrCl_4_ is not a particularly stable form of Zr and reacts with water indicating that this is perhaps a transient Zr form.

Of the NM properties, zeta potential and in turn, ion exchange capacity, play the most significant role in NM–soil interactions. Clay and organic matter are negatively charged so the positively charged Ce_0.75_Zr_0.25_O_2_ is likely to have been held in the soil matrix, potentially forming interactions with PO_4_^3−^ and NO_3_^−^. This could form part of its mechanism, improving lettuce biomass through slow release of essential nutrients like PO_4_^3−^ and NO_3_^−^, without increasing N emissions. This is particularly relevant post transformation, as CeO_2_ has been shown to heteroaggregate with soil clay particles and other natural soil colloids.^52^ There is a lack of CePO_4_ seen in the experimental soil samples, which indicates that any CeO_2_–PO_4_^3−^ interaction would be through weaker forces such as van der Waal forces in larger agglomerates rather than *via* chemical bonding. Positively charged NMs also interact differently with plant roots than negatively charged ones, typically remaining on the outside of root surfaces.^5,53,54^ CeO_2_ NMs surface charge can be modulated through application of PO_4_^3−^ ions, acting to neutralise and change the zeta potential of the NMs, encouraging translocation into aboveground plant tissues.^55,56^ The negatively charged ZSM-5-15 is capable of forming interactions with soil cations, using ion exchange to prevent soil pH reduction while promoting nitrification through release of NH_4_^+^. The binding dynamics between ZSM-5-15 and BEA-19 are clearly different, despite the similarities in their compositions, Si/Al ratio, zeta potential and agglomerate size. Both zeolites are bound to NH_4_^+^ and have similar pore sizes, of 6.8 and 5.5 Å for BEA-19 and ZSM-5-15, respectively. With two zeolites of such great similarity, it is hard to determine how they have such different effects on soil N cycling, leachate emissions and lettuce growth on the basis of this dataset alone.

### 4.4. Ce_0.75_Zr_0.25_O_2_ and ZSM-5-15 positively impact lettuce growth with maintained crop quality

ZSM-5-15 is able to increase both lettuce biomass and N content, however the impact of ZSM-5-15 on N_2_O emissions means that it cannot be considered as a sustainable alternative to conventional fertilisation. BEA-19 improves the micronutrient status of lettuce but has a negative impact on lettuce biomass accumulation. This is suggested to be due to the very low lettuce growth seen under BEA-19 treatment, which may be the result of a deficiency in Zn.^57^ The Zn concentration is much higher in the control lettuce, with the other treatments producing lettuces all within a similar range for Zn concentration in shoot tissue (0.00011–0.00021 mg g^−1^). Control lettuce biomass was much lower than all other treatments, indicating that the control lettuces' stunted growth was due to a lack of available nitrogen and phosphate in particular, which led to a greater but still deficient Zn concentration.^58^ NPK full treatment acidified the soil; this resulting change in soil pH is the most probable cause of the decreased biomass accumulation, with the NPK half treatment having a similar soil pH at week 8 to the control treatments, with resulting greater lettuce biomass accumulation than in the NPK full treatment. Ce_0.75_Zr_0.25_O_2_ treatment promotes lettuce biomass accumulation, without impacting on soil N emissions. However, the NM fails to improve the crop's quality, with no significant differences to any other treatments for macro- or micronutrient tissue concentrations. Therefore, the ratio of nutrients to biomass remains consistent and there is greater lettuce biomass under NM treatments, leading to maintenance of lettuce's quality in terms of nutritive content, partnered with improved yield. Under alkaline conditions, as in all treatments other than NPK full, the increased N content of soil is linked to increased Zn, Cu, Fe and Mn uptake. However, increased P fertiliser additions may have a negative effect on micronutrient uptake, having been shown to affect Zn among others.^59^

None of the lettuce tissues measured displayed high MDA content, a key marker of lipid peroxidation in response to reactive oxygen species production during oxidative stress. MDA concentration was only recorded after destructive sampling so transient changes in MDA concentration over the plants' growing period were not studied. Slightly elevated MDA concentrations were found for NPK full and ZSM-5-15 grown lettuce. This may be due to the more acidic soil found under NPK full treatment, while the effect of ZSM-5-15 on lettuce MDA levels may be due to the action of the NM itself, *i.e.*, a physical effect. It could be through this minor stress that greater biomass accumulation was seen, as many nanofertilisers act to improve crop growth through initiation of minor stress, triggering improved crop stress tolerance through prior activation of stress signalling pathways.^60^

Nano-CeO_2_ has been shown to have an antioxidative effect, mimicking the antioxidative enzymes catalase (CAT) and superoxide dismutase (SOD) until binding with phosphate.^61,62^ From the Ce L3-edge XANES spectra linear combination fitting it is clear that there is very limited binding of Ce to PO_4_^3−^ in the soil environment, indicating that there is potential for CeO_2_ to be acting as a nanozyme, promoting lettuce growth and improving crop stress resilience. However, how the heteroagglomerates of CeO_2_ with associated soil anions, clay particles and colloids behave, and how these other compounds are able to influence nanozyme activity is currently unknown.

### 4.5. Outlook

ZSM-5-15 treatment drives changes in the soil N cycling, most notably through increasing N_2_O emissions. This is theorised to be through the action of ZSM-5-15 NMs on nitrifying microbes, likely be aided through its supplementation of NH_4_^+^ ions as part of its structure. This theory is supported by the soil respiration and leachate nutrient emissions data, in particular NH_4_^+^ and NO_3_^−^ emissions. Disentangling the mechanism of ZSM-5-15 action on N_2_O emissions requires further study. Stable isotope ^15^N labelling of NO_3_^−^ and NH_4_^+^ would be required to determine whether N_2_O is being produced *via* nitrification or denitrification pathways. If the increase in N_2_O from ZSM-5-15 is through nitrification then qPCR analysis of relevant N-cycling genes in bacteria, but also archaeal and fungal groups, would elucidate how ZSM-5-15 may be affecting the soil microbial community. Further research on shifts in microbial communities upon NM application in conjunction with fertilisers would also aid understanding of potential ecotoxicological impacts of the NMs on soil microbes. Additionally, deciphering what zeolite characteristics trigger the effect would enable improved design and agricultural application of other zeolites also. There are undetermined factors that also influence the differences in NM effect, for example the most significant differences between BEA-19 and ZSM-5-15 zeolite NMs remains unclear in terms of their effect on crop growth and soil N cycling dynamics.

Ce_0.75_Zr_0.25_O_2_ NMs provide a promising avenue for future research, with increased biomass accumulation without triggering increased gaseous or leachate emissions, or soil acidification, whilst also maintaining the lettuce's micronutrient content with this increased biomass accumulation. The mixed metal oxide is likely leaching Zr^4+^ ions, leading to the formation of ZrCl_4_, Ce_0.9_Zr_0.1_O_2_ and CeO_2_, which then act independently in the soil and on the plant. The NMs' zeta potential, particularly relating to ion exchange capacity, interaction with soil colloids, anions and cations, are all important factors in NM activity in soils. The impact of the leached Zr^4+^ ions on soil microorganisms is highly dependent on what compounds it subsequently forms, with ZrCl_4_ previously shown to impact the reproduction of model terrestrial species *Enchytraeus crypticus*.^63^ As such, further research is required to fully determine the broader ecological impacts of Ce_0.75_Zr_0.25_O_2_ NMs release into agroecosystems, especially the extent to which NM transformation products impact different soil species.

NMs transform as they enter the soil, with further transformations inside plant tissues.^64^ Understanding how the transformed NMs go on to interact with plant roots and if further transformations occur at the plant root surface due to root exudates or inside plant tissues is yet to be understood for Ce_0.75_Zr_0.25_O_2_, ZSM-5-15 and BEA-19. Just as these NMs are transforming, understanding how these changes alter their agglomeration and binding dynamics is important to reveal how they behave in natural systems. For example, visualising heteroagglomerates would aid in developing more detailed knowledge around NM kinetics, realistic agglomerate size and interactions with biotic soil components. NM heteroaggregates have been studied under lab conditions using scanning transmission electron microscopy (STEM), but methods for complex environmental sample heteroagglomerates and compound identification is lacking.^65–67^ To understand how Zr compounds and CeO_2_ act in the soil post transformation from Ce_0.75_Zr_0.25_O_2_, these visualisations or other characterisation methods in complex samples are required.

## Author contributions

Jessica Chadwick: writing – original draft, investigation, formal analysis, visualisation, conceptualization, funding acquisition. Aleksandar Radu: resources, writing – review and editing. Iuliia Mikulska: formal analysis, visualisation, funding acquisition, writing – review and editing. Swaroop Chakraborty: investigation, funding acquisition, writing – review and editing. Peng Zhang: supervision, funding acquisition. Sami Ullah: conceptualization, supervision, writing – review and editing, funding acquisition. Iseult Lynch: conceptualization, supervision, writing – review and editing, funding acquisition.

## Conflicts of interest

There are no conflicts to declare.

## Supplementary Material

EN-012-D5EN00526D-s001

## Data Availability

Supplementary information (SI): statistical test results, lettuce nutrient concentrations and XANES spectra of experimental samples and reference standards. See DOI: https://doi.org/10.1039/D5EN00526D. The data supporting this article have been included as part of the SI.
