# Supplementary material for: Soil-based zeolite and metal oxide nanomaterial application alters reactive nitrogen losses and lettuce (Lactuca sativa L.) growth
Source: Environ Sci Nano. 2025 Sep 26;12(11):4966–79. doi: 10.1039/d5en00526d (PMC12495385; doi:10.1039/d5en00526d)
Supplement: EN-012-D5EN00526D-s001 [file EN-012-D5EN00526D-s001.pdf]

Supplementary Information (SI) for Environmental Science: Nano.
This journal is © The Royal Society of Chemistry 2025

Soil-based zeolite and metal oxide nanomaterial application alters reactive Nitrogen losses and lettuce (*Lactuca sativa L.*) growth

Jessica J. Chadwick,[1] Iuliia Mikulska,[2] Aleksandar Radu,[3] Swaroop Chakraborty,[1] Peng Zhang,[1] Sami Ullah,[1] Iseult Lynch[1]**

[1] School of Geography, Earth and Environmental Sciences, University of Birmingham, Edgbaston, Birmingham B15 2TT, UK
[2] Diamond Light Source Ltd, Harwell Science & Innovation Campus, Didcot, Oxfordshire OX11 0DE, UK
[3] School of Chemistry, University of Lincoln, Lincoln LN6 7TS, UK

* Corresponding authors: Jessica J. Chadwick, jessicajchadwick@gmail.com; Iseult Lynch, i.lynch@bham.ac.uk

Supplementary Information

Table S1. *Greenhouse water physicochemical analyses. Values displayed are means and standard error of the mean. EC: Electrical conductivity.*

	**pH**	**EC (µS cm^{-1}) (n = 3)**	**Ammonia (mg N L^{-1}) (n = 3)**	**Nitrite (mg N L^{-1}) (n = 3)**	**Nitrate (mg N L^{-1}) (n = 3)**	**Phosphate (mg P L^{-1}) (n = 3)**
**Greenhouse water**	7.34 (±0.03)	205 (±1.2)	0.0294 (± 0.007)	0.0003 (± 0.0006)	0.695 (± 0.001)	0.504 (± 0.014)

Additional methodological details for acid digestion of lettuce and soil samples

Detection of the uptake of nanomaterials was performed by inductively coupled plasma optical emission spectroscopy (ICP-OES). For this, 0.5g of dry lettuce tissue or dried soil per treatment was weighed. Lettuce tissue was dissolved in 10 mL of nitric acid (HNO_3), and soils were dissolved with 9 mL HNO_3 and 3 mL hydrofluoric acid (HF). Lettuce digestions occurred at 210˚C for 15 minutes in a MARS 6 microwave digestion system in which iWave temperature sensors were floor-mounted to detect the solution temperature during digestion, while the soils were digested at 180˚C for 10 minutes using the same set-up. The respective digestion program details are available in Table S1 and S2.

Table S2. *Program details for Mars 6 Microwave Digester heat-assisted digestion of lettuce tissue using 0.5g dry tissue with 10mL HNO3 as sourced from Mars 6 Microwave Acid Digestion Method Note Compendium.*

Parameter	Setting
**Temperature (˚C)**	210
**Ramp (mm:ss)**	15:00
**Hold (mm:ss)**	15:00
**Pressure (psi)**	800
**Power (W)**	1000

Table S3. *Program details for Mars6 Microwave Digester heat-assisted digestion of soil using 0.5g dry soil with 9mL HNO_3 and 3mL HF as based on US EPA 3052 via Mars 6 Microwave Acid Digestion Method Note Compendium.*

Parameter	Setting
**Temperature (˚C)**	180
**Ramp (mm:ss)**	05:30
**Hold (mm:ss)**	09:30
**Pressure (psi)**	800
**Power (W)**	1000

Additional methodological details for elemental analysis of C, N and S

Soil samples were analysed using a FlashSmart Elemental analyser (Thermo Scientific, USA) using a CHNS method with the furnace at 950˚C using vanadium pentoxide as a combustion aid. Lettuce samples were analysed for C and N using a vario PYRO cube (Elementar Analysensysteme GmbH, Hanau, Germany) in combustion mode at 950˚C and then a reduction tube at 600˚C. A thermal conductivity detector was used for signal detection.

Additional methodological details for X-ray absorption spectroscopy

The energy of the storage ring during the data collection was 3.0 GeV, with the current intensity of 300 mA. A Si(111) double crystal monochromator was used to select the incident X-ray energy. Pt-coated collimating and focusing mirrors were used to collect the spectra. A pair of dedicated Pt-coated mirrors operated at 7.0 mrad angle was used to eliminate high energy harmonics during the Ce L_{III} - edge spectra measurements. Zr K-edge XAS spectra were measured without harmonic rejection mirrors. A set of reference compounds, consisting of cerium and zirconium, including $CePO_4$, $Ce(CH_3COO)_3$, CeO_2, $CeCl_4$, Zr metal, $ZrCl_4$ and $ZrO(NO_3)_2$ were measured in transmission mode using three consecutive ionization chambers, where the reference samples were placed between the first and second ionization chambers. In addition, a range of different ratios of Ce/Zr mixed metal oxide NMs and ZrO_2 reference compounds were measured in fluorescence mode using a Vortex-ME7 silicon drift detector with the Xspress3X digital pulse processor. Powdered reference compounds ($CePO_4$, $Ce(CH_3COO)_3$, $ZrCl_4$ and $ZrO(NO_3)_2$) were mixed with boron nitride (BN) and pressed into pellets. 3000 mg L^{-1} NMs dispersed in water reference compounds (different ratios of Ce/Zr mixed metal oxide NMs, ZrO_2 and CeO_2) were measured in liquid cells with thickness of 1 mm. Energy calibration of collected spectra was achieved by simultaneous measurements of Zr K-edge and Cr K – edge XAS spectra on zirconium and chromium reference foils for Zr K-edge and Ce L_{III} -edge measurements, correspondingly. Spectra on reference foils were

measured in transmission mode. The reference foils were placed between the second and third ionization chambers. Spectra from two to six repetitions for each sample were merged to improve signal-to-noise ratio.

Results

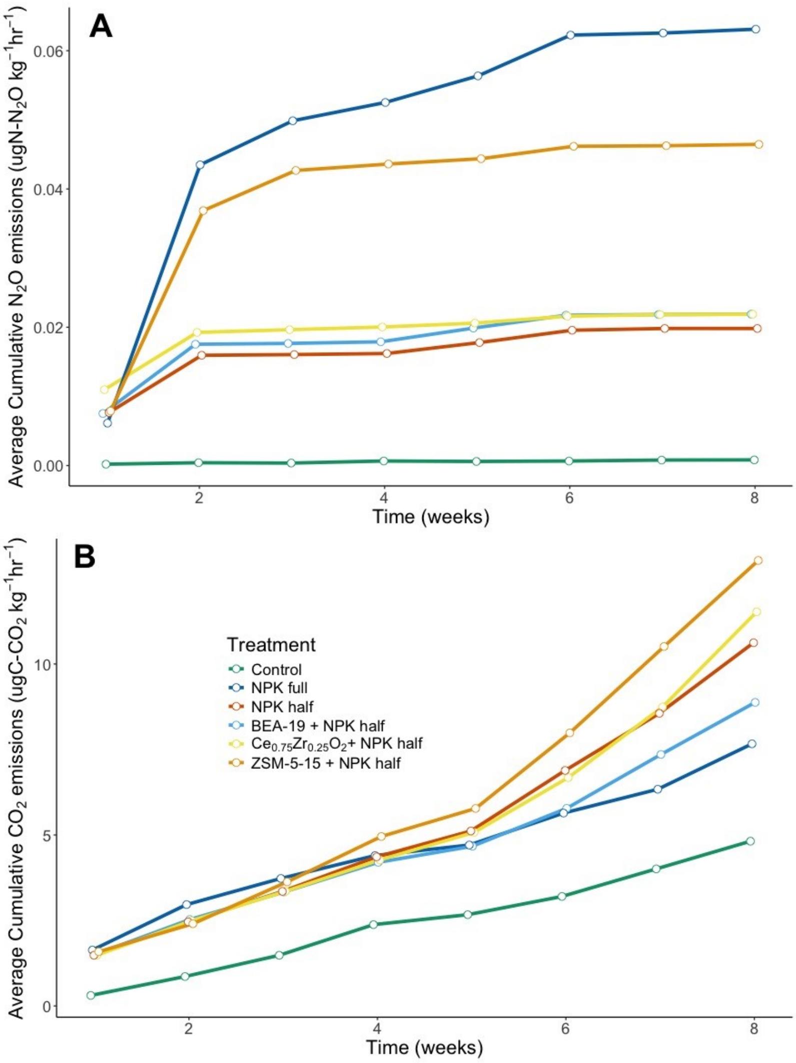

***Figure S*1.** *Cumulative emissions from soil of N_2O (A) and CO_2 (B) over the course of the 8-week growing period. The details of the treatments and their associated colours (for both panels A and B) are shown as the inset in panel B. NPK full is the standard application of nitrogen, phosphorus and potassium (180kg/hm^2 N in the form of urea and 200kg/ hm^2 in the form of potassium phosphate monobasic), while NPK half is a half-dose of each (90kg/hm^2 N and 100kg/hm^2 P_2O_5 and K_2O). Control is no fertiliser at all, and no nanomaterials. The nanomaterial treatments are all with NPK half treatment. Nanomaterial treatments include BEA-19and ZSM-5-15 two nano-zeolites, and $Ce_{0.75}Zr_{0.25}O_2$, a nano-metal oxide.*

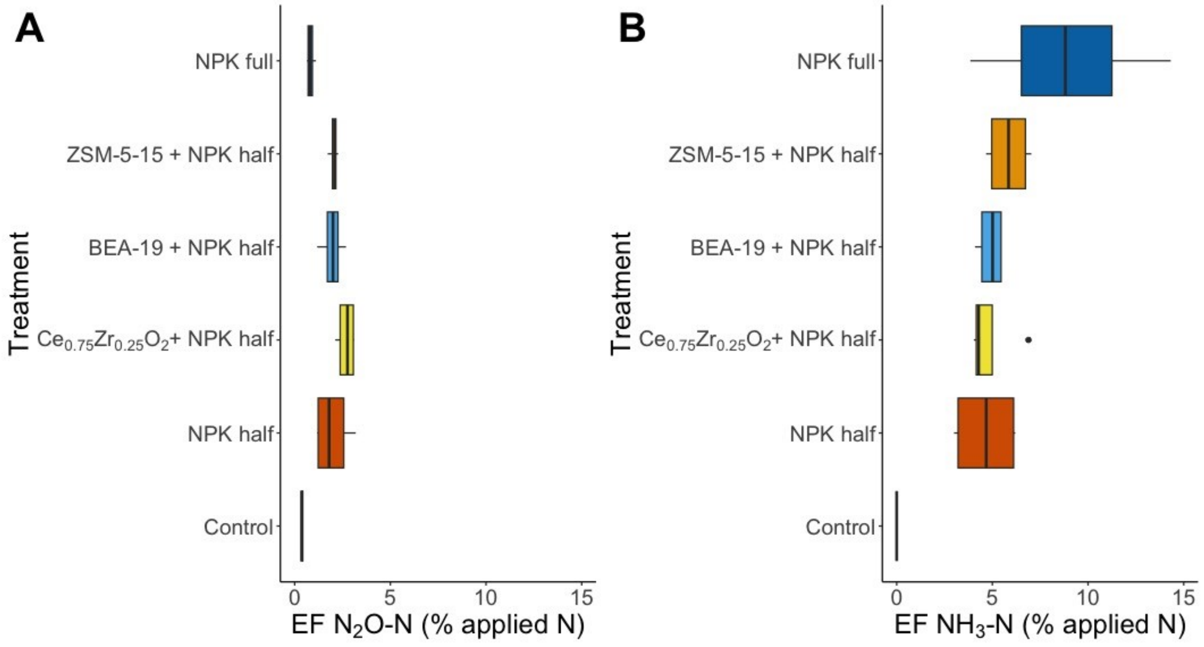

Figure S2. *Emission factors of N_2O (A) and NH_3 (B) gas emissions as a proportion of the total N added to each treatment. NPK full is the standard application of nitrogen, phosphorus and potassium (180kg/ha N in the form of urea and 200kg/ha in the form of potassium phosphate monobasic), while NPK half is a half-dose of each (90kg/ha N and 100kg/ha P_2O_5 and K_2O). Control is no fertiliser at all, and no nanomaterials. The nanomaterial treatments are all with NPK half treatment. Nanomaterial treatments are BEA-19and ZSM-5-15 two nano-zeolites. $Ce_{0.75}Zr_{0.25}O_2$ is a nano-metal oxide.*

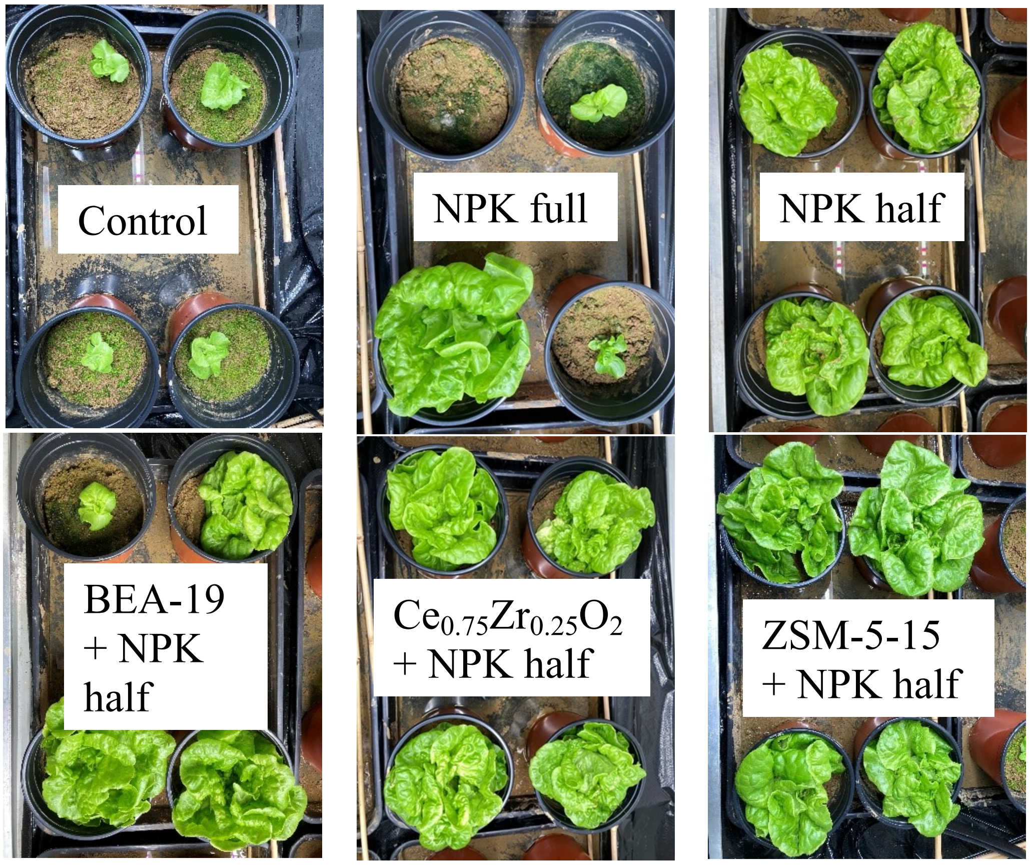

Figure S3. *Images of matured lettuce heads for each treatment at week 8 of the greenhouse experiment prior to destructive tissue sampling.*

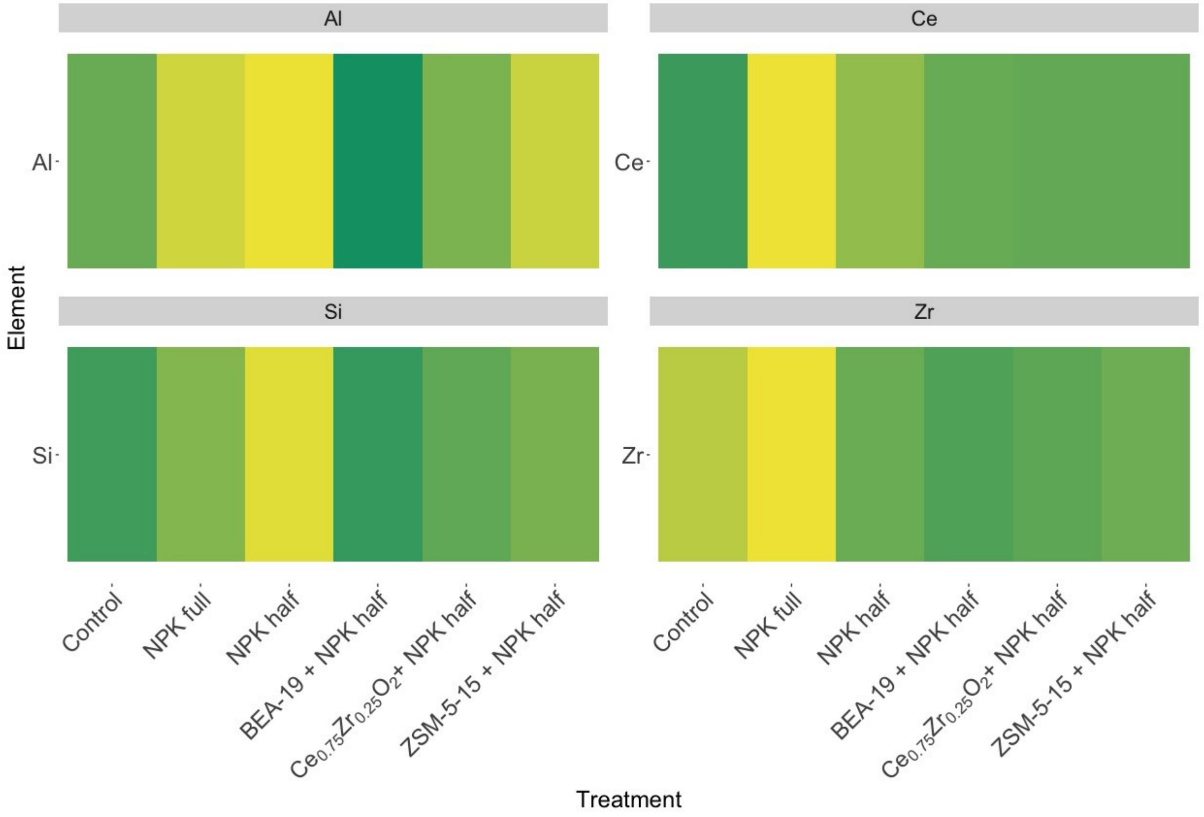

Figure S4. *Relative concentration of elemental constituents of NMs used in $Ce_{0.75}Zr_{0.25}O_2$, BEA-19 and ZSM-5-15 treatments found in lettuce tissue compared across the six treatments. BEA-19 and ZSM-5-15 are zeolites containing Al and Si. NPK full is the standard application of nitrogen, phosphorus and potassium (180kg/ha N in the form of urea and 200kg/ha in the form of potassium phosphate monobasic), while NPK half is a half-dose of each (90kg/ha N and 100kg/ha P_2O_5 and K_2O). Control is no fertiliser at all, and no nanomaterials. The nanomaterial treatments are all with NPK half treatment. Numerical concentration data available in Table S4.*

Table S4*. P-values of statistically significant differences in mineralised NO_3^- concentration of different treatments in relation to NPK full treated soil. NPK full is the standard application of nitrogen, phosphorus and potassium (180kg/ha N in the form of urea and 200kg/ha in the form of potassium phosphate monobasic), while NPK half is a half-dose of each (90kg/ha N and 100kg/ha P_2O_5 and K_2O). Control is no fertiliser at all, and no nanomaterials. The nanomaterial treatments are all with NPK half treatment.*

	**Control**	**NPK full**	**NPK half**	**BEA-19 + NPK half**	**$Ce_{0.75}Zr_{0.25}O_2$ + NPK half**	**ZSM-5-15 + NPK half**
**Mineralised NO_3^- (mg kg^{-1} dry soil) (n=4)**	0.00021	-	0.0034	0.011	0.00064	0.00025

Table S5. *P-values of statistically significant differences in mean CO_2 gaseous emissions of different treatments in relation to control soil and lettuce. NPK full is the standard application of nitrogen, phosphorus and potassium (180kg/ha N in the form of urea and 200kg/ha in the form of potassium phosphate monobasic), while NPK half is a half-dose of each (90kg/ha N and 100kg/ha P_2O_5 and K_2O). Control is no fertiliser at all, and no nanomaterials. The nanomaterial treatments are all with NPK half treatment.*

	Control	NPK full	NPK half	BEA-19 + NPK half	$Ce_{0.75}Zr_{0.25}O_2$ + NPK half	ZSM-5-15 + NPK half
**Mean CO_2 emissions ($\mu g\ kg^{-1}\ h^{-1}$) (n=4)**	-	0.0785	<0.0001	0.0055	<0.0001	<0.0001

Table S6*. P-values of statistically significant differences in leachate NO_3^- concentration of different treatments in relation to the control soil. NPK full is the standard application of nitrogen, phosphorus and potassium (180kg/ha N in the form of urea and 200kg/ha in the form of potassium phosphate monobasic), while NPK half is a half-dose of each (90kg/ha N and 100kg/ha P_2O_5 and K_2O). Control is no fertiliser at all, and no nanomaterials. The nanomaterial treatments are all with NPK half treatment.*

	Control	NPK full	NPK half	BEA-19 + NPK half	$Ce_{0.75}Zr_{0.25}O_2$ + NPK half	ZSM-5-15 + NPK half
**NO_3^- (mg L^{-1} leachate) (n=4)**	-	0.044	0.00395	-	0.0056	0.0386

Table S7. *Concentration of elemental nutrients across shoot lettuce tissue grown in the six different treatment soils. NPK full is the standard application of nitrogen, phosphorus and potassium (180kg/ha N in the form of urea and 200kg/ha in the form of potassium phosphate monobasic), while NPK half is a half-dose of each (90kg/ha N and 100kg/ha P_2O_5 and K_2O). Control is no fertiliser at all, and no nanomaterials. The nanomaterial treatments are all with NPK half treatment.*

	Control (N=4)	NPK full (n=3)	NPK half (N=4)	BEA-19 + NPK half (N=4)	$Ce_{0.75}Zr_{0.25}O_2$ + NPK half (N=4)	ZSM-5-15 + NPK half (N=4)
**Ca (mg/g)**	0.15746843	0.18306005	0.18378503	0.15617002	0.16820275	0.15144748
**Cu (mg/g)**	0.03080826	0.02429536	0.02592205	0.02117193	0.02624364	0.02643565
**Fe (mg/g)**	0.02605645	0.00946766	0.01781764	0.01873396	0.02013273	0.01119552
**Mn (mg/g)**	0.00070856	0.00048091	0.00058748	0.00051019	0.00059444	0.00038113
**Mg (mg/g)**	0.03080826	0.02429536	0.02592205	0.02117193	0.03375122	0.02643565
**Zn (mg/g)**	0.00056602	0.00015337	0.00011078	0.00012825	0.0001721	0.00020532
**P**	0.02046859	0.03596641	0.02301406	0.0192665	0.02012262	0.02425014

(mg/g)						
**K (mg/g)**	0.44987047	0.49895419	0.37169321	0.30797222	0.27396367	0.297656
**Ce (mg/g)**	8.8761E-05	3.1517E-06	5.4653E-05	7.3909E-05	0.00016856	5.6479E-05
**Zr (mg/g)**	1.0457E-05	-	3.8643E-05	2.4673E-05	2.9459E-05	3.4717E-05
**Si (mg/g)**	0.01307812	0.01017922	0.00985731	0.00858166	0.00987787	0.01204843
**Al (mg/g)**	0.02370997	0.00900661	0.01746708	0.01797256	0.01906906	0.0108446
**N (%)**	3.55643	4.06831333	4.807775	4.807695	4.456745	5.2650925

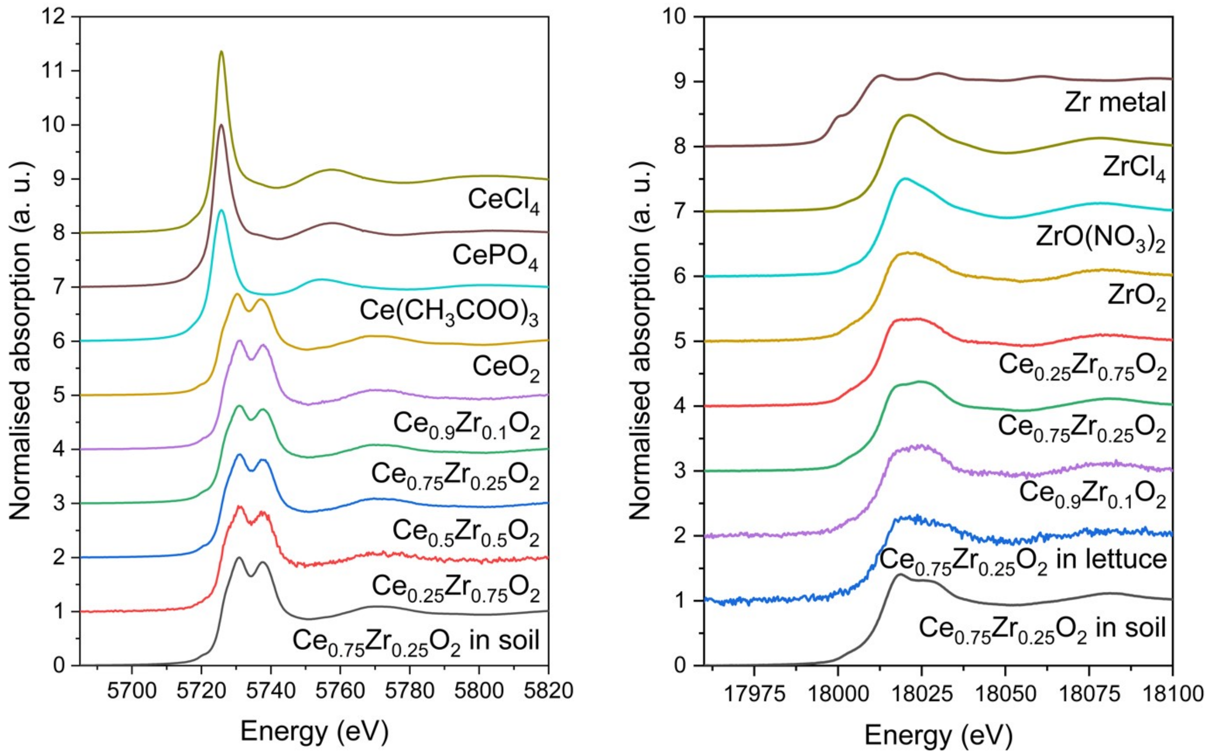

Figure S5. *(A) Ce L3-edge XANES spectra measured on experimental soil samples with added* $Ce_{0.75}Zr_{0.25}O_2$ *NMs and NPK half and Ce-containing reference standards. (B) Zr K-edge XANES spectra measured on experimental soil samples with added* $Ce_{0.75}Zr_{0.25}O_2$ *NMs and NPK half and lettuce grown in soil and Zr-containing reference standards.*
